# Supplementary figures and images for: Effects of different amoxicillin treatment durations on microbiome diversity and composition in the gut
Source: PLoS One. 2022 Oct 27;17(10):e0275737. doi: 10.1371/journal.pone.0275737 (PMC9612567; doi:10.1371/journal.pone.0275737)

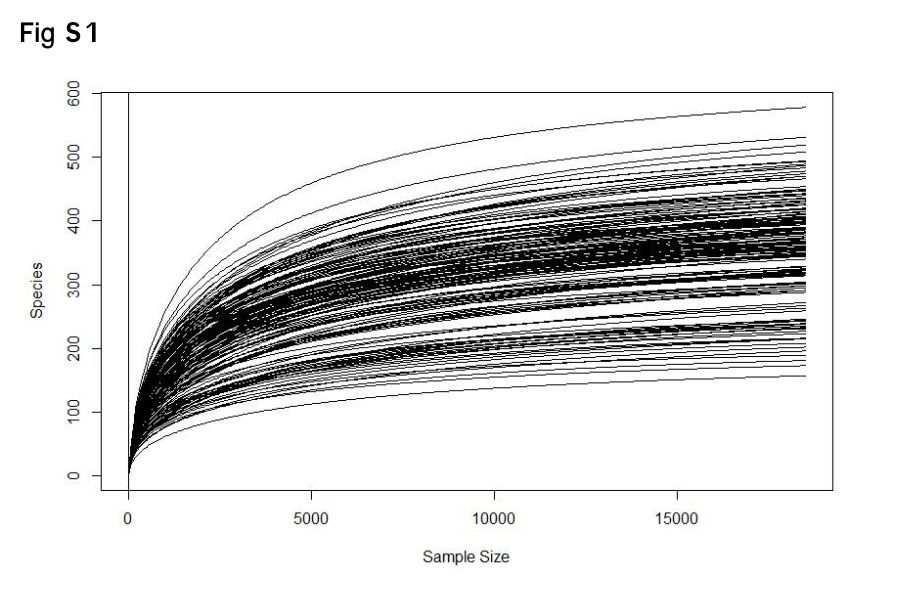

Supplement: S1 Fig — (TIF) [file pone.0275737.s001.tif]

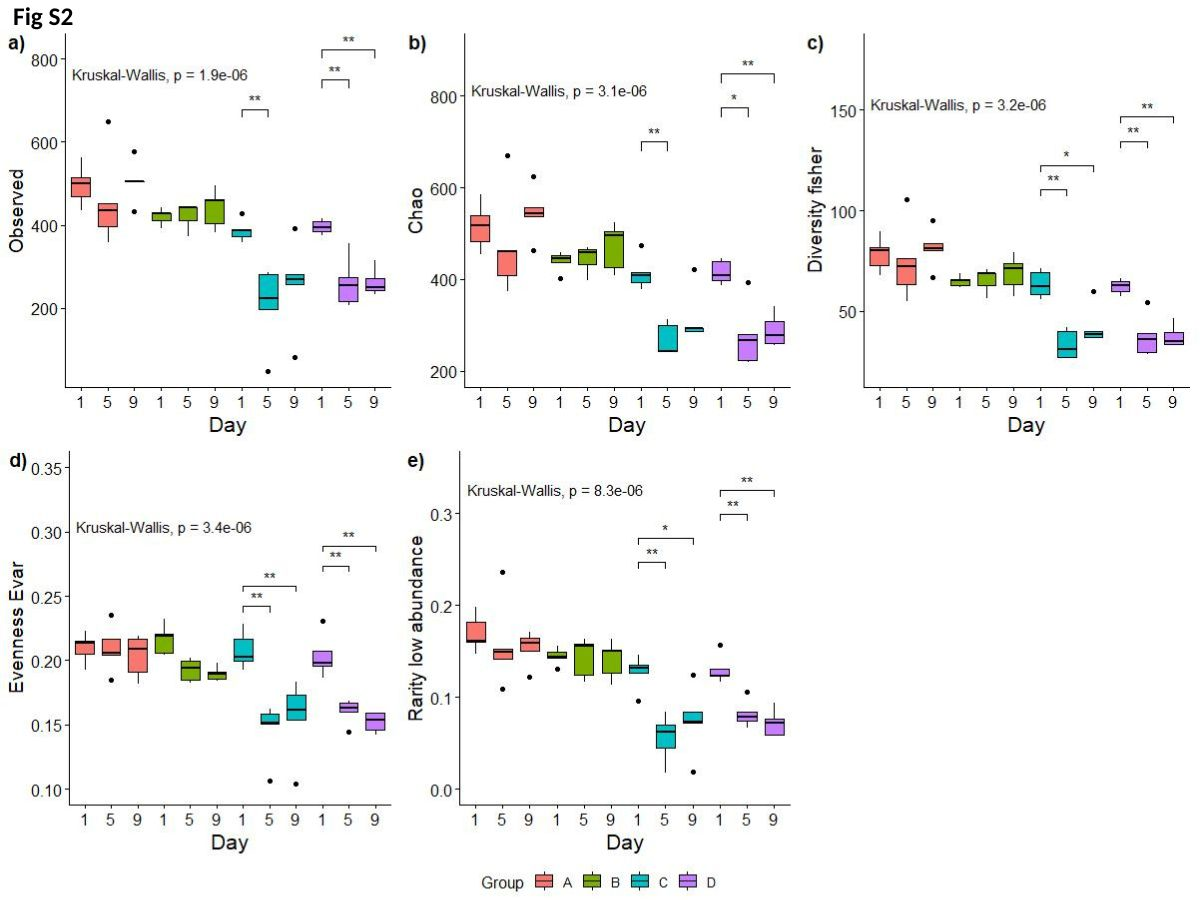

Supplement: S2 Fig — Observed number of ASVs, richness (Chao1), diversity (Fisher) and evenness (Evar) and Rarity low abundance indexes observed in mice treated with amoxicillin for 0 days (control group A), 3 days (group B), 7 days (group C) and 14 days (group D), during antibiotic treatment (day 1–9 of the experiment). The x-axis shows the group (A/B/C/D) and sampling-number (1 = day 1, 2 = day 5, 3 = day 9). Boxes indicate interquartile range (IQR) between the first and third quartiles (25th and 75th percentiles respectively), and the horizontal line inside the box defines the median. Whiskers represent the lowest and highest values within 1.5 times the IQR from the first and third quartiles, respectively. p-values to Kruskal–Wallis test is designated on the figure and symbols * = p < 0.05, ** = p < 0.01, *** = p < 0.001, according to Wilcoxon test. (TIF) [file pone.0275737.s002.tif]

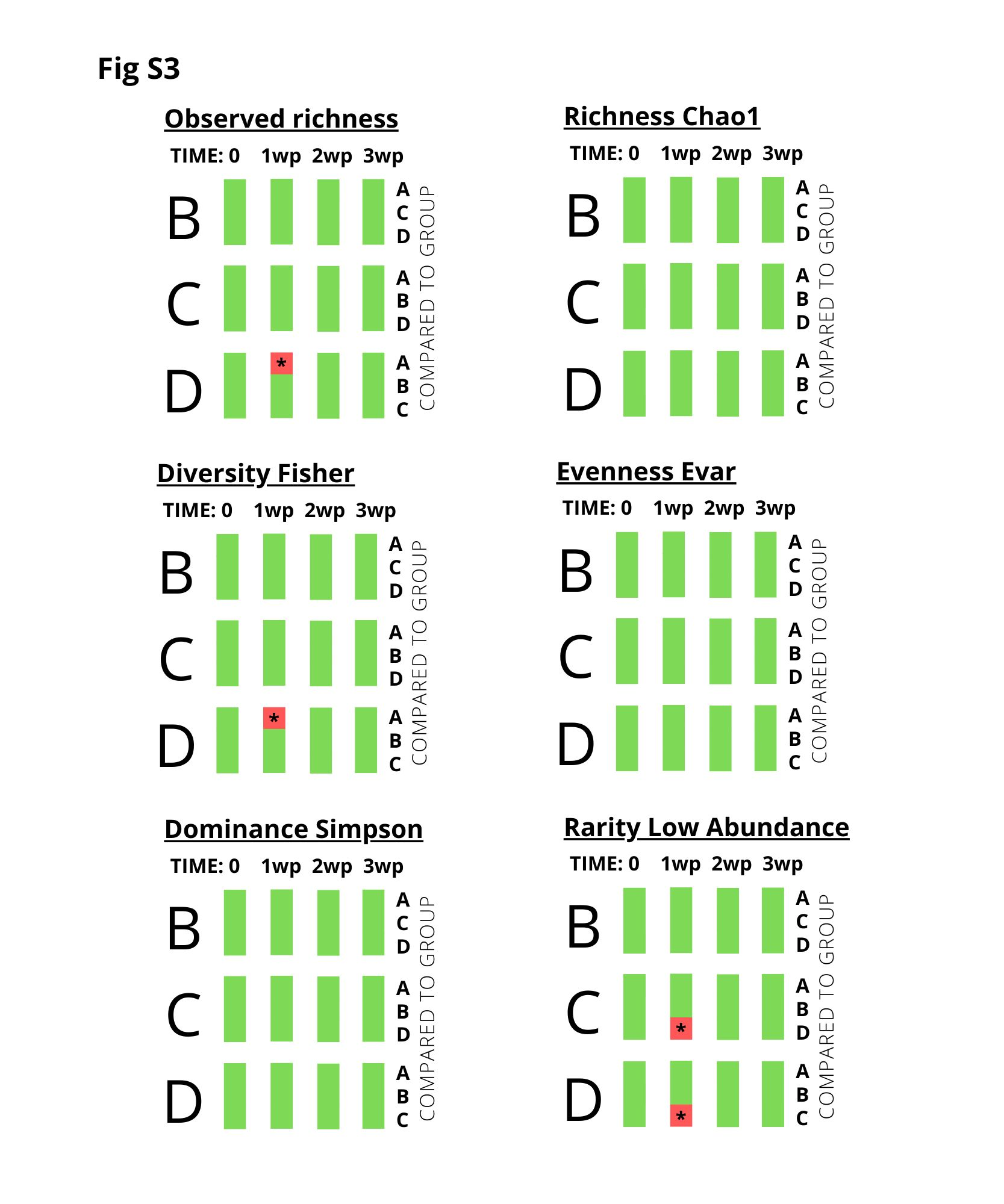

Supplement: S3 Fig — Comparison of the following indexes: Observed richness, Chao1, Fisher diversity, evenness Evar, Dominance Simpson, Rarity low abundance and dominance core abundance, for mice treated with amoxicillin for 0 days (A), 3 days (B), 7 days (C) and 14 days (D), before intake of amoxicillin (Time = 0), 1 week past end of antibiotic treatment (Time = 1wp), 2 weeks past end of antibiotic treatment (Time = 2wp), 3 weeks past end of antibiotic treatment (Time = 3wp). Colours indicates: Grey = no significant difference, red = significant difference; * = p < 0.05, ** = p < 0.01). (TIF) [file pone.0275737.s003.tif]

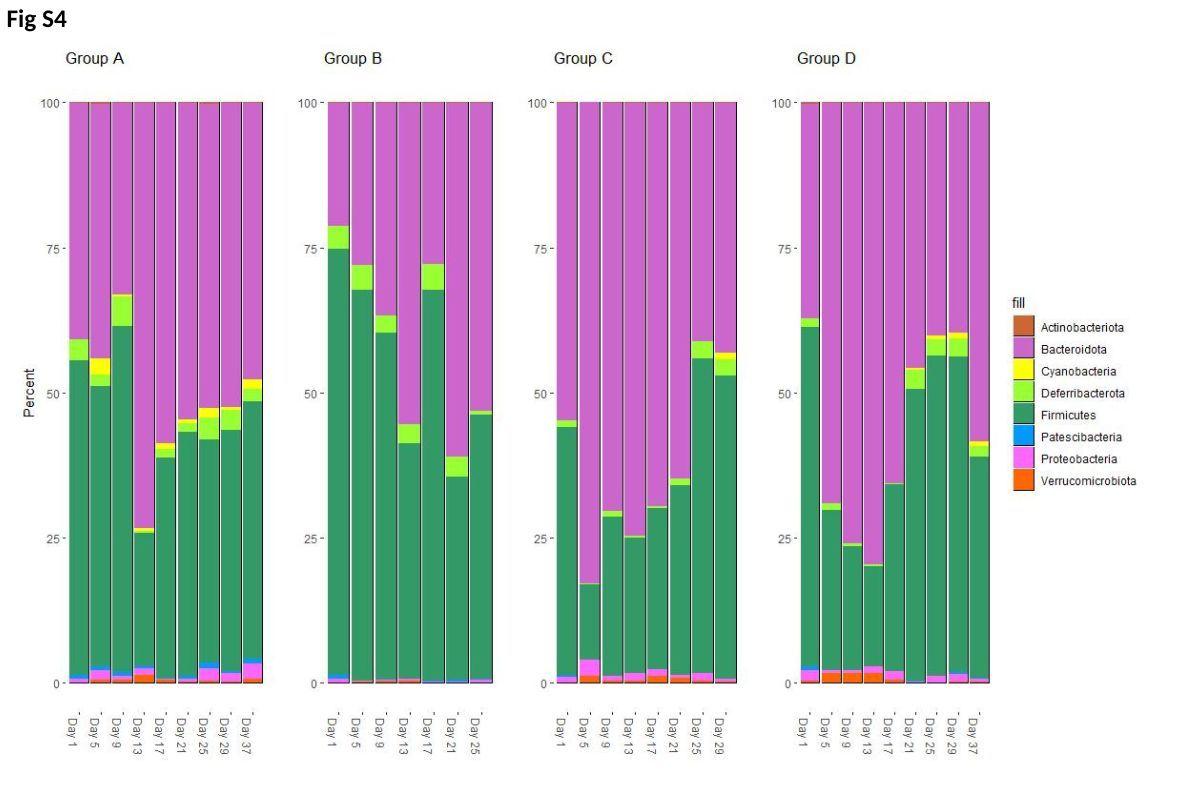

Supplement: S4 Fig — Different coloured bars represent different phyla (as listed in the legend) and sampling day is indicated on the x-axis. (TIF) [file pone.0275737.s004.tif]

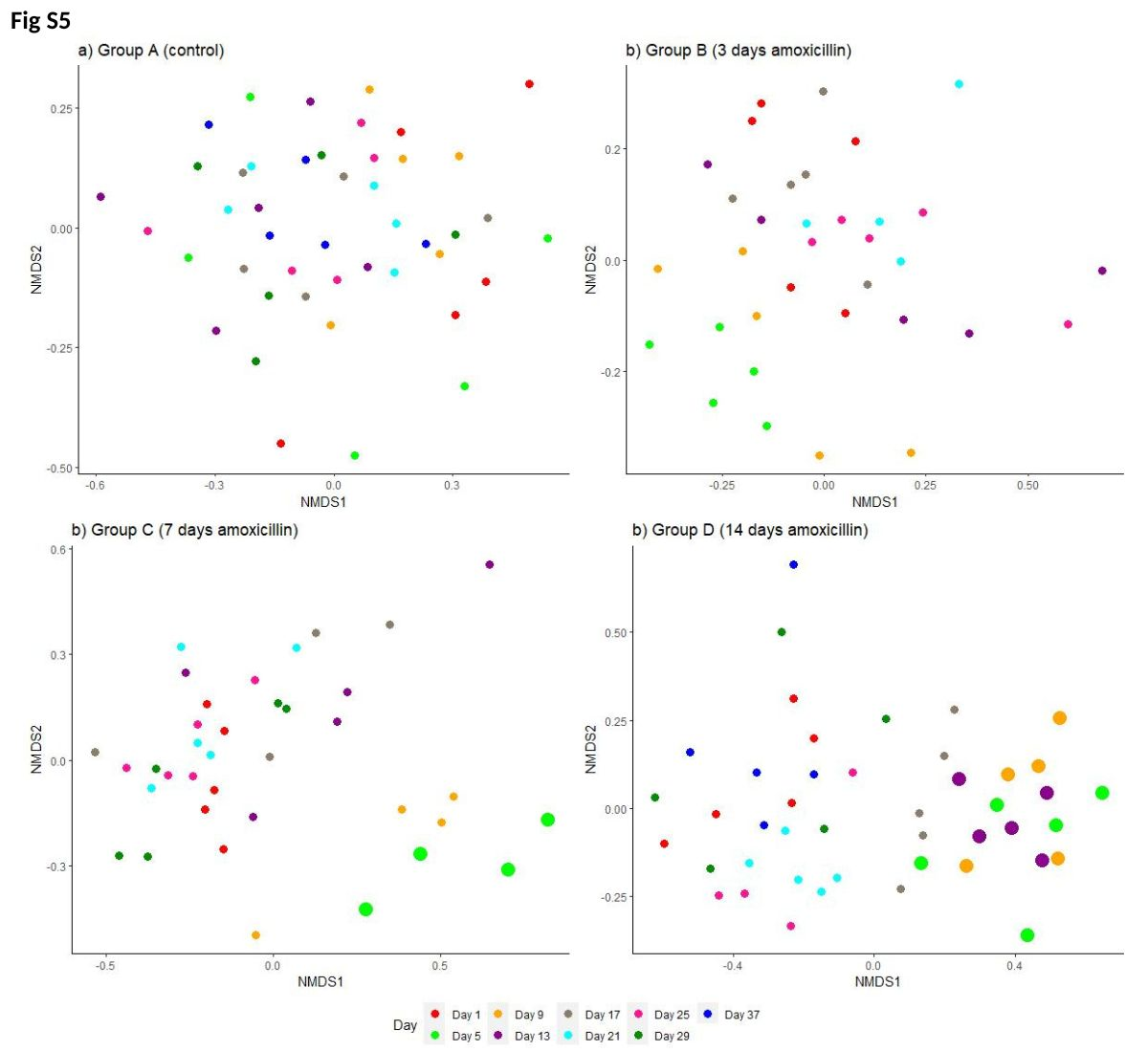

Supplement: S5 Fig — Non-metric multidimensional scaling (NMDS) ordination plot based on Bray-Curtis dissimilarity of community composition, of all samples from mice treated with amoxicillin for a) 0 days (control, group A), b) 3 days (group B), c) 7 days (group C), and d) 14 days (group D). The colour of the dots indicates sampling day as described in the legend in the diagram, and samples taken during antibiotic treatment are indicated by increased size. (TIF) [file pone.0275737.s005.tif]

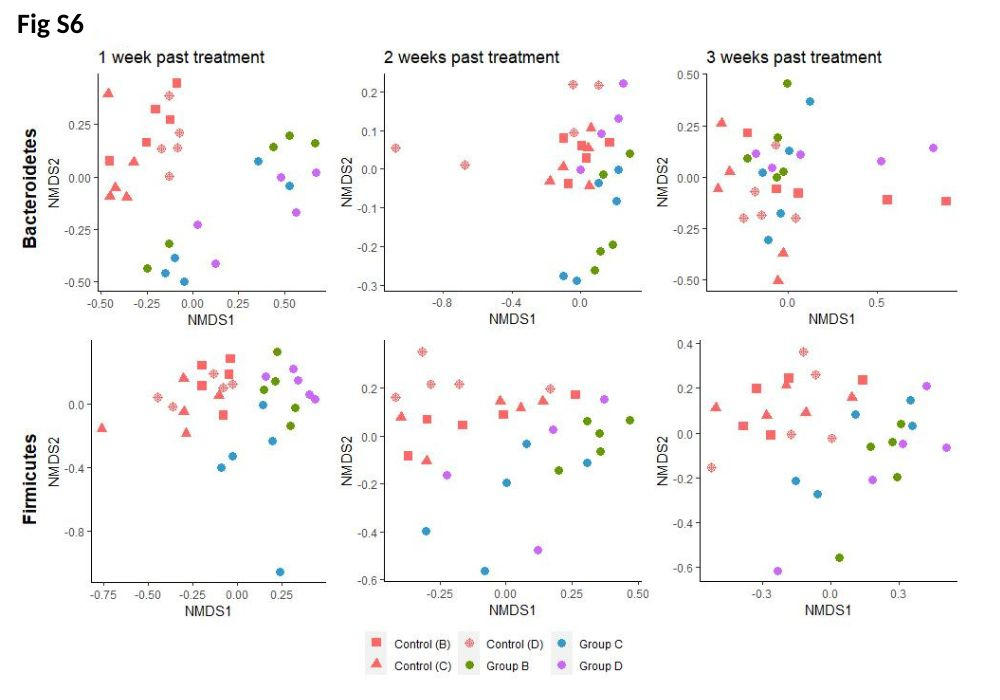

Supplement: S6 Fig — Non-metric multidimensional scaling (NMDS) ordination plot based on Bray-Curtis dissimilarity of Firmicutes and Bacteriodetes community composition, including samples collected from all four treatment groups, 1, 2 and 3 weeks after end of treatment with amoxicillin. The colour of the dots indicates treatment group (red = control group A, green = group B receiving amoxicillin for 3 days, blue = group C receiving amoxicillin for 7 days, group D receiving amoxicillin for 14 days). (TIF) [file pone.0275737.s006.tif]

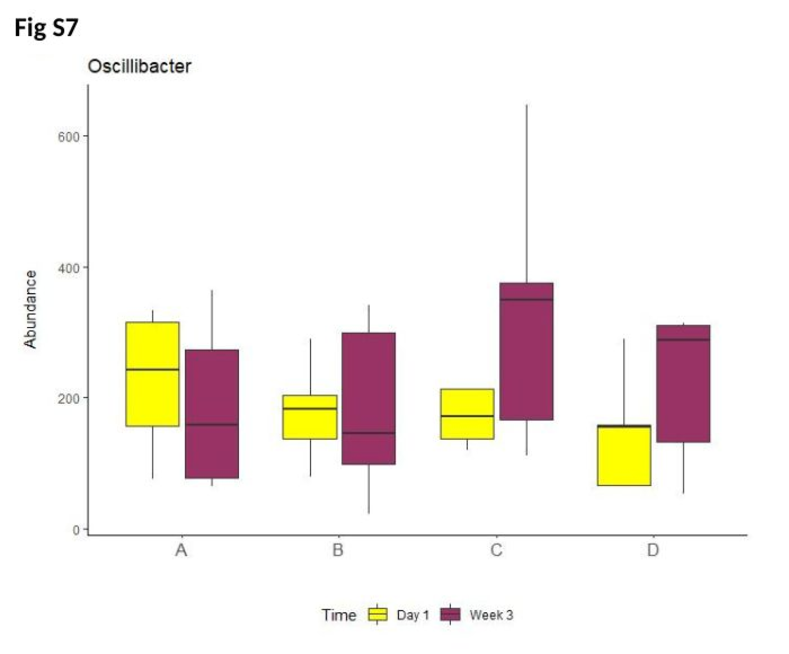

Supplement: S7 Fig — Boxes indicate interquartile range (IQR) between the first and third quartiles (25th and 75th percentiles respectively), and the horizontal line inside the box defines the median. Whiskers represent the lowest and highest values within 1.5 times the IQR from the first and third quartiles, respectively. (TIF) [file pone.0275737.s007.tif]
