# Supplementary material for: Effects of different amoxicillin treatment durations on microbiome diversity and composition in the gut
Source: PLoS One. 2022 Oct 27;17(10):e0275737. doi: 10.1371/journal.pone.0275737 (PMC9612567; doi:10.1371/journal.pone.0275737)
Supplement: S1 Table — (DOCX) [file pone.0275737.s008.docx]

| Sample name | Raw sequences | After quality check |
| --- | --- | --- |
| A1FS1 | 52179 | 45407 |
| A1FS2 | 45434 | 38324 |
| A1FS3 | 57424 | 40590 |
| A1FS4 | 54980 | 46506 |
| A1FS5 | 55752 | 44665 |
| A1FS6 | 86516 | 54942 |
| A1FS7 | 74594 | 50193 |
| A1FS8 | 53000 | 46840 |
| A1FS10 | 67908 | 44406 |
| A2FS1 | 56047 | 37348 |
| A2FS2 | 39506 | 33059 |
| A2FS3 | 59880 | 40957 |
| A2FS4 | 50714 | 38403 |
| A2FS5 | 54604 | 44676 |
| A2FS6 | 74735 | 59415 |
| A2FS7 | 70482 | 52666 |
| A2FS8 | 68332 | 44420 |
| A2FS10 | 56163 | 51001 |
| A3FS1 | 60465 | 47737 |
| A3FS2 | 34318 | 28535 |
| A3FS3 | 55137 | 35434 |
| A3FS4 | 58401 | 37672 |
| A3FS5 | 56736 | 39518 |
| A3FS6 | 72844 | 63586 |
| A3FS7 | 78117 | 59604 |
| A3FS8 | 84882 | 58533 |
| A3FS10 | 65396 | 58252 |
| A4FS1 | 46680 | 41714 |
| A4FS2 | 35503 | 29570 |
| A4FS3 | 53166 | 43699 |
| A4FS4 | 60235 | 41253 |
| A4FS5 | 50179 | 40817 |
| A4FS6 | 73150 | 50814 |
| A4FS7 | 75294 | 63450 |
| A4FS8 | 71885 | 66969 |
| A4FS10 | 65999 | 58082 |
| A5FS1 | 61101 | 48238 |
| A5FS2 | 66911 | 49031 |
| A5FS3 | 56667 | 44399 |
| A5FS4 | 55737 | 35255 |
| A5FS5 | 50460 | 39173 |
| A5FS6 | 71823 | 46874 |
| A5FS7 | 69091 | 58299 |
| A5FS8 | 65834 | 45729 |
| A5FS10 | 62237 | 51780 |
| B1FS1 | 51828 | 44374 |
| B1FS2 | 65942 | 42385 |
| B1FS3 | 51022 | 37819 |
| B1FS4 | 56300 | 38808 |
| B1FS5 | 55339 | 35168 |
| B1FS6 | 64191 | 50515 |
| B1FS7 | 67706 | 62104 |
| B2FS1 | 57374 | 45769 |
| B2FS2 | 62467 | 42665 |
| B2FS3 | 51188 | 37901 |
| B2FS4 | 47511 | 43678 |
| B2FS5 | 50247 | 39151 |
| B2FS6 | 73873 | 52040 |
| B2FS7 | 79459 | 55804 |
| B3FS1 | 51112 | 43453 |
| B3FS2 | 59934 | 38255 |
| B3FS3 | 53070 | 41489 |
| B3FS4 | 52640 | 42919 |
| B3FS5 | 62222 | 36879 |
| B3FS6 | 67694 | 56357 |
| B3FS7 | 69776 | 50414 |
| B4FS1 | 53433 | 44372 |
| B4FS2 | 65493 | 43202 |
| B4FS3 | 62668 | 45249 |
| B4FS4 | 49077 | 43105 |
| B4FS5 | 52903 | 34754 |
| B4FS7 | 63843 | 46185 |
| B5FS1 | 43895 | 34093 |
| B5FS2 | 64700 | 42331 |
| B5FS3 | 63228 | 46451 |
| B5FS4 | 52646 | 41512 |
| B5FS5 | 50424 | 41539 |
| B5FS6 | 69917 | 53041 |
| B5FS7 | 68350 | 42757 |
| C1FS1 | 29930 | 18529 |
| C1FS2 | 61650 | 46966 |
| C1FS3 | 68660 | 42363 |
| C1FS4 | 52045 | 40492 |
| C1FS5 | 51030 | 37932 |
| C1FS6 | 69488 | 56196 |
| C1FS7 | 69299 | 52189 |
| C1FS8 | 76293 | 53447 |
| C2FS1 | 38591 | 34877 |
| C2FS2 | 58502 | 46904 |
| C2FS3 | 64570 | 44299 |
| C2FS4 | 60590 | 39147 |
| C2FS5 | 49184 | 39797 |
| C2FS6 | 70641 | 52490 |
| C2FS7 | 73556 | 47830 |
| C2FS8 | 85069 | 52532 |
| C3FS1 | 34329 | 32278 |
| C3FS2 | 59210 | 40401 |
| C3FS3 | 67205 | 40419 |
| C3FS4 | 59772 | 45462 |
| C3FS6 | 58827 | 50897 |
| C3FS7 | 62721 | 43524 |
| C3FS8 | 67887 | 53439 |
| C4FS1 | 40548 | 34743 |
| C4FS2 | 58406 | 40107 |
| C4FS3 | 67368 | 44286 |
| C4FS4 | 62046 | 40012 |
| C4FS5 | 64147 | 45474 |
| C4FS6 | 63216 | 51413 |
| C4FS7 | 69402 | 59539 |
| C4FS8 | 68800 | 62777 |
| C5FS1 | 30624 | 28673 |
| C5FS2 | 53840 | 40364 |
| C5FS3 | 62711 | 45240 |
| C5FS4 | 64480 | 41970 |
| C5FS5 | 79603 | 51083 |
| C5FS6 | 70234 | 53151 |
| C5FS7 | 65360 | 54514 |
| C5FS8 | 81607 | 54769 |
| D1FS1 | 36468 | 33004 |
| D1FS2 | 49491 | 43794 |
| D1FS3 | 66186 | 51278 |
| D1FS4 | 60145 | 38316 |
| D1FS5 | 76073 | 50104 |
| D1FS6 | 69284 | 44336 |
| D1FS7 | 60795 | 53527 |
| D1FS10 | 60305 | 47639 |
| D2FS1 | 46431 | 35641 |
| D2FS2 | 57183 | 37611 |
| D2FS3 | 62774 | 41203 |
| D2FS4 | 61861 | 48114 |
| D2FS5 | 80083 | 49505 |
| D2FS6 | 65357 | 41676 |
| D2FS7 | 55973 | 52105 |
| D2FS8 | 62851 | 50278 |
| D2FS10 | 71163 | 51675 |
| D3FS1 | 44284 | 36249 |
| D3FS2 | 55905 | 44215 |
| D3FS3 | 53401 | 40672 |
| D3FS4 | 55801 | 41596 |
| D3FS5 | 77093 | 56712 |
| D3FS6 | 65226 | 46311 |
| D3FS7 | 64759 | 53173 |
| D3FS8 | 69336 | 44754 |
| D3FS10 | 68164 | 51654 |
| D4FS1 | 50613 | 36023 |
| D4FS2 | 56354 | 39379 |
| D4FS3 | 56212 | 45392 |
| D4FS4 | 63342 | 46770 |
| D4FS5 | 84883 | 51726 |
| D4FS6 | 84731 | 61312 |
| D4FS7 | 75741 | 56006 |
| D4FS8 | 64341 | 48280 |
| D4FS10 | 58112 | 44201 |
| D5FS1 | 47830 | 40423 |
| D5FS2 | 61121 | 43904 |
| D5FS3 | 58992 | 37060 |
| D5FS4 | 60098 | 44988 |
| D5FS5 | 86687 | 57188 |
| D5FS6 | 76418 | 50700 |
| D5FS7 | 65237 | 45127 |
| D5FS8 | 78534 | 52005 |
| D5FS10 | 63046 | 50487 |
