# Supplementary material for: Effects of different amoxicillin treatment durations on microbiome diversity and composition in the gut
Source: PLoS One. 2022 Oct 27;17(10):e0275737. doi: 10.1371/journal.pone.0275737 (PMC9612567; doi:10.1371/journal.pone.0275737)
Supplement: Attachment — Submitted filename: Response to reviewers.pdf [file pone.0275737.s012.pdf]

Dear Editor PLOS ONE,

We have submitted a manuscript to PLOS ONE with the title 'Effects of different amoxicillin treatment durations on microbiome diversity and composition in the gut' with submission ID: PONE-D-21-37884. The manuscript is a revised version of a previous manuscript submission made by the same authors to PLOS ONE spring 2021 with the submission ID: PONE-D-21-12675. The submission PONE-D-21-12675 was rejected by PLOS ONE after a review process. We were offered to make an appeal, and submit a new version within 2 weeks. However, the manuscript need to undergo substantial changes so we decided to make a new submission.

We have now added new data and made major changes to the manuscript based on the reviewers comments from the first submission (submission ID: PONE-D-21-12675), where we found it appropriate. We have attached a version of the manuscript with Track-changes and the appeal form. Below you will find our point-by-point comments to the reviewers from our previous submission ID: PONE-D-21-12675.

. The following styles are used:

- The Reviewers original comments as is.

- *Our answers to the comments are indicated in italics.*

Thanks for re-reviewing our manuscript. We hope the improvements and our responses are satisfactory and we hope our manuscript will be re-considered for publication in PLOS ONE.

With kind regards and for the Authors,

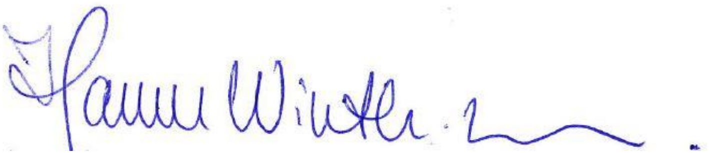

Hanne C. Winther-Larsen

PONE-D-21-12675
Dear Editor
The full title is: Effects of different amoxicillin treatment durations on bacterial diversity in the gut
PLOS ONE

Dear Dr. Winther-Larsen,

Thank you for submitting your manuscript to PLOS ONE. After careful consideration, we have decided that your manuscript does not meet our criteria for publication and must therefore be rejected.

Specifically:

All three reviewers noted considerable concerns with the quality and rigor of the study. There appears to be a serious technical flaw in the study design and resulting outcomes that undermines any conclusions that can be drawn. The authors may wish to reconsider the use of amoxicillin for the study. The impact of antibiotic treatment on gut microbiota generally is much more substantial than what is indicated here, where the changes are barely above the noise level. In addition, the organization and presentation of the data needs considerable work, as does the English editing.

I am sorry that we cannot be more positive on this occasion, but hope that you appreciate the reasons for this decision and that the reviewer comments may assist you in considering your continued efforts in this area.

Yours sincerely,

Brenda A Wilson, Ph.D.
Academic Editor
PLOS ONE

[Note: HTML markup is below. Please do not edit.]

Reviewers' comments:

Reviewer's Responses to Questions

Comments to the Author

1. Is the manuscript technically sound, and do the data support the conclusions?

The manuscript must describe a technically sound piece of scientific research with data that supports the conclusions. Experiments must have been conducted rigorously, with appropriate controls, replication, and sample sizes. The conclusions must be drawn appropriately based on the data presented.

Reviewer #1: No

Reviewer #2: Partly

Reviewer #3: Yes

We have made major changes to the manuscript.

---

2. Has the statistical analysis been performed appropriately and rigorously?

Reviewer #1: Yes

Reviewer #2: No

Reviewer #3: Yes

We have made major changes to the manuscript.

____

3. Have the authors made all data underlying the findings in their manuscript fully available?

The PLOS Data policy <http://www.plosone.org/static/policies.action#sharing> requires authors to make all data underlying the findings described in their manuscript fully available without restriction, with rare exception (please refer to the Data Availability Statement in the manuscript PDF file). The data should be provided as part of the manuscript or its supporting information, or deposited to a public repository. For example, in addition to summary statistics, the data points behind means, medians and variance measures should be available. If there are restrictions on publicly sharing data—e.g. participant privacy or use of data from a third party—those must be specified.

Reviewer #1: Yes

Reviewer #2: No

Reviewer #3: Yes

We will make the data available in a depository when the manuscript is accepted for publication.

____

4. Is the manuscript presented in an intelligible fashion and written in standard English?

PLOS ONE does not copyedit accepted manuscripts, so the language in submitted articles must be clear, correct, and unambiguous. Any typographical or grammatical errors should be corrected at revision, so please note any specific errors here.

Reviewer #1: Yes

Reviewer #2: No

Reviewer #3: Yes

All authors have thoroughly revised the manuscript.

____

5. Review Comments to the Author

Please use the space provided to explain your answers to the questions above. You may also include additional comments for the author, including concerns about dual publication, research ethics, or publication ethics. (Please upload your review as an attachment if it exceeds 20,000 characters)

Reviewer #1: The manuscript is not in standard English and needs editing. However, it is very understandable and clear.

We appreciate the reviewers comment that the manuscript is very understandable and clear. All authors have in additions thoroughly revised the manuscript.

The overall effects of antibiotic treatment on the gut microbiota are generally very profound, with PCoA plots and bar diagrams showing community compositions that are altered to an extent far greater than those seen in this study.
For example, Graversen and colleagues (Front. Microbiol., 26 March 2020 | https://doi.org/10.3389/fmicb.2020.00496) saw more than 50% of the community structure of the microbiota in laboratory rats change as a result of treatment with amoxicillin.
In mice, Castro-Mejía and colleagues (Research in Veterinary Science, Volume 118, June 2018, Pages 357-364) saw similar dramatic changes following treatment of mice with ampicillin, which has the same anti-microbial effects as ampicillin.
In general, exposure to a wide range of antibiotics cause in an almost complete change in community composition. (Front Cell Infect Microbiol. 2019; 9: 99.) These changes are invariably much more pronounced on PCoA plots than the changes observed in this study.

The reviewer is concerned that there must be a flaw with our study design as others have published a larger impact on the microbiome than what we report. In the three published papers that the reviewer exemplifies the sampling time is either at a shorter time-point after antibiotic treatment has ceased (after one day, where we also see a strong effect) (Graversen et al., 2020), used different antibiotics and treated the mice for altogether three weeks (Sun et al., 2019), or used a sterile environment (Castro-Mejia et al., 2018) which was compared to non-sterile conditions which showed a totally different impact on the microbiome. We suspect that different antibiotics, treatment lengths and post-sampling time-points will all influence the results which is what we have shown in our study. It should also be emphasized that several studies investigating the effects of amoxicillin conclude that overall diversity and composition is reversed relatively soon after exposure to amoxicillin ceases (Elvers 2020, Antonopoulos 2009, Panda 2014), however, that some taxa-specific alterations in lower phylogenetic levels may be preserved for longer (Palleja 2018). In our study we demonstrated significant impacts on overall diversity and composition during exposure to antibiotics, and significant effect in certain specific taxa which was preserved up to 3 weeks after termination of the treatment.

Unfortunately, the authors need to address the possibility that their antibiotic treatment was unsuccessful. Amoxicillin is difficult to administer reliably via drinking water, and ampicillin, which has a similar anti-microbial spectrum, is generally preferred for experimental purposes. (Injection is much more reliable than administration via drinking water.) It seems less likely, but is it possible that the animals had been previously exposed to a penicillin-family antibiotic, which could have led to an adapted and resistant microbiota?

The reviewer suggested that we should have inject amoxicillin in the mice than supply it in the drinking water. We disagree with the reviewer on this point. We are, in this study, using mice as a model for potential effects of use of amoxicillin in humans. As amoxicillin is an oral antibiotic so it makes little sense to inject the drug. Moreover, amoxicillin is recommended at three daily doses. Hence injection the drug three time a day into the mice to obtain a level of antibiotic above MIC is an inappropriate study design. The reviewer also referred to three published papers as examples of influence of antibiotics on the gut microbiome, where in fact the antibiotic where given orally in all three studies (Graversen et al., 2020, Castro-Mejia et al., 2018, Sun et al., 2019 as stated above). Reviewer 1 concern that the mice had received antibiotics previously is pure speculation and we can ensure the reviewer that this is not the case. As our data demonstrate, there are significant effects from the amoxicillin during treatment, which do not indicate an adapted and resistant microbiota.

Reviewer #2: The manuscript “Effects of different amoxicillin treatment durations on bacterial diversity in the gut” by Lekang et al evaluated the broad changes in the fecal microbiota induced by

different durations of amoxicillin treatment. The authors concluded that a longer treatment duration of amoxicillin led to a longer recovery time for the gut microbiota. This is a complicated study, but this manuscript is not well-organized and written to clarify the details. Audience needs to keep guessing the meaning or intentions of the authors to understand the manuscript. Figure legends need to be improved to clarify the details of each figure (please see examples below).

We have major changes to the manuscript with these comments in mind. Please see document Manuscript with Track-changes

Major concerns:

1. Were the food, water and bedding material sterile? 14 days treatment of amoxicillin should have significantly tipped the balance of microbiota even at phylum level if the environment is sterile.

The reviewer suggest that we should have performed the study in a sterile environment. In our opinion that would also have been an interesting study, however, if we try to mimic a natural conditions during antibiotics treatment, mice nor humans leave in a sterile environment. We will all be exposed to bacteria before, during and after antibiotic treatments.

2. Based on the description, “day 1” used in figure 1 and throughout the manuscript should be changed to “day 0”. Therefore, 3 days treatment of amoxicillin will be from day 0 to day 3. In figure 1, please clarify that day 0 is prior to amoxicillin treatment. The current schematic is confusing.

We have clarified in that day 1 refers to the first day of the experiment and that samples collected on this were taken prior to adding amoxicillin to the water.

3. What are the changes at genus level? As discussed in line 403-410, genus changes are more affected by antibiotics. The authors should evaluate changes at genus level to make the conclusion if gut microbiota recovered or not after antibiotics treatment.

We agree with the reviewer that higher resolution data will increase the interest and significance of the manuscript. Therefore, we have added data describing changes of higher resolution in the gut microbiome after amoxicillin treatment. Please see additions described in Figure 6 and 7, as well as line 444-469, 500-501 and 506-537 in the discussion.

4. For line 222-234, the authors should include a brief description of how 37% (line 225) was calculated. I assume that 37% was calculated by 420 divided by the sum of numbers in figure 3a Day1. Therefore, it would be clear to state: 37% (420 out of xxx). In addition, the resolution of all the figures is too low, and I was not able to download a high resolution figure to see the number, either.

We agree with the reviewer that this information should be provided in the manuscript and have added information in this section (line 259-267). Further, we have improved several figures by modifying the font-size.

5. Line 224. “no significant difference”. The authors should define what is considered significant in this case, and any statistic method was used to conclude no significant difference?

We agree with the reviewer that this is confusing and have changed this to "The results demonstrated minor differences in the common core microbiome…" (line 255).

Minor:

1. Introduction, line 67. It should be cardiovascular disease based on the reference.

This has been changes in the new version of the manuscript. Please see line 75.

2. Materials and methods, sample preparations and sequencing, line 153. The kit should be for 600bp.

Read length is 300 bp as stated in the manuscript.

3. Line 214. Figure S1. Please define the full name for "observed".

As described in the figure text, "Observed" indicate the <u>*number of observed ASVs*</u>*, and we consider this as sufficient information in the figure itself.*

4. Line 290. In addition

This paragraph has been rewritten. Please see line 243-259.

5. Line 331 and Figure 3b. Please make sure the phylum names are scientifically standardized.

This has been changes to Bacteroidetes, consistently through the manuscript

6. For figure 4, the abbreviated labeling should be defined in the figure legend. For example, 3 days AB indicates the group of mice that received 3 days of amoxicillin treatment, or to be consistent with figure 1, this group can be labeled as group B, etc. My assumption is that (figure 4a) day 1 means samples were collected on day 1 (actually day 0). However, in figure 4b, 4c and 4d, I still can't figure out what is the meaning of control 1*, control 2* and control 3*. What is the meaning of "*" also?

This is reperesented by Figure 5 in the updated version of the manuscript and the information have been clarified.

Reviewer #3: The manuscript is interesting and the experiment is well designed. Data are presented in a transparent manner. Statistical analysis is performed appropriately. English is fine, however some improvements should be made.

We thank the reviewer for this comment. We have now carefully through the manuscript to improve the English language.

____

6. PLOS authors have the option to publish the peer review history of their article (what does this mean? <https://journals.plos.org/plosone/s/editorial-and-peer-review-process#loc-peer-review-history>). If published, this will include your full peer review and any attached files.

If you choose “no”, your identity will remain anonymous but your review may still be made public.

Do you want your identity to be public for this peer review? For information about this choice, including consent withdrawal, please see our Privacy Policy <https://www.plos.org/privacy-policy> .

Reviewer #1: No

Reviewer #2: No

Reviewer #3: No

[NOTE: If reviewer comments were submitted as an attachment file, they will be attached to this email and accessible via the submission site. Please log into your account, locate the manuscript record, and check for the action link "View Attachments". If this link does not appear, there are no attachment files.]

- - - - -
For journal use only: PONEDEC3

____

In compliance with data protection regulations, you may request that we remove your personal registration details at any time. (Remove my information/details) <https://www.editorialmanager.com/pone/login.asp?a=r> . Please contact the publication office if you have any questions.

Dear authors,

The study is interesting and well designed, figures provide clear data. However, some improvements need to be implemented to lift up the scientific value of this paper.

We thank the reviewer for this comment.

Please take under consideration the following remarks.

Introduction:

L 61: Please provide numbers. Specify the number of bacterial cells in human body. Provide refence for this statement.

Since estimates regarding the number of cells vary greatly, we have changed this to "More than half of all cells inside the human body are bacteria" and added a reference.

L.69-71: This part should me mentioned before the part discussing the role of human microbiome.

This has been changed in the current version of the manuscript. Factors affecting the microbiome is described in line 69-72, and health effects in line 73-75.

L 73: Please provide active substances with broad antimicrobial spectrum.

This have been provided in line 78.

L 74: Word "inhabit" is not correct. Should be "exhibit" or "display".

This have been changed to display, see line 80.

L 82-L85: Various results of microbiom alterations are listed. I recommend to put this paragraph together with L65-68; as obesity is a consequence of deregulated metabolism. Please provide the possible consequences of microbiome alterations in one paragraph. Describe clearly the cause and effect.

We have modified this, however, possible effects of microbiome disturbance in general (line 74-75) and effects of antibiotic treatments (line 92-95) is still written separately, since we consider this to be clearer for a new reader.

L93: Please specify "Longer". Is it prolonged treatment or longer than what?

This has been specified (line 104-105).

You mentioned that changes in the microbiome may lead to resistance development. But it is necessary to underlie that cessation of antibiotic treatment earlier than prescribed (e.g. 7-day treatment prescribed but the patient take only 5 days) is a cause of increasing resistance as well. (L93)

While several studies have demonstrated a higher risk of resistance development with longer treatment lengths, there has to our knowledge been no clinical studies comparing treatment lengths, demonstrating increased risk of AMR development among patients receiving treatments with shorter treatment length. We have further stated that the implementation of shorter treatments is in several cases regarded as safe and this has been recommended as a standard procedure against certain

infections. It is important to note that shorter treatment lengths it is not implemented for all antibiotics, and doctors' advice and prescriptions should always be followed by the patients.

Adequate change should be made in Abstract.

Major changes has been made to the Abstract

L 94-97:Please specify in what kind of infection it is reasonable to shorten antibiotic treatment. Please also list antibiotics used in such cases

Information regarding infections with possible shorter antimicrobial treatment lengths have been added (line 109-111)

L 104: Please provide a reference for the statement that amoxicillin is a first choice antibiotic for treating mentioned infections. Refer to EUCAST, CLSI, EMA recommendations or other.

This has been added (line 121)

Please add an information that amoxicillin is frequent combined with β-lactamases inhibitor like clavulanic acid. Describe briefly, if some research discuss the influence of such a combination on gut microbiome. If so, it would be valuable to compare Your results with those obtained from such an experiment in Discussion section.

This is now included in the discussion (line 538-545)

Material and methods:

L:118: Please provide a reference for given dosing regimen.

This has been added (line 147)

L 120: What kind of fruit flavor was used? What Substance? Did You eliminated the fact that this ingredient may had its own effect in gut microbiome?

It is widely known that certain food additives (both synthetic and natural) have an impact on gut microbiome diversity. Please, describe it in Experimental consideration section to avoid speculation that microbiome alteration may had appeared not only because of amoxicillin treatment.

This has been added to the discussion (line 569-575)

Results:

Line 219: "However, two weeks past treatment, there were no statistical difference between group D and the control group."

Amoxicillin as a time-dependent antibiotic with no post-antibiotic effect. Microbiome alterations lasts 2 weeks after treatment cessation in a group where amoxicillin was administered 14 days. If 2 weeks is enough to rebuilt microbiome, why to pursue to shorten the treatment in case of antibiotics with time-dependent effect? Are benefits will sill exceed the risk?

Studies investigating effects on gut microbiomes, from e.g. antibiotics, diet etc, commonly find that the overall diversity and composition is reversed to original state relatively fast after ceasing of the treatment investigated, as here, while abundance of certain specific taxa may stay affected for prolonged time. In this version of the manuscript we have conducted a more thorough investigation of taxa at lower phylogenetic levels.

L240-251: In case of group B some exceptions occurred in day 17. Please try to make a hypothesis what may be the cause of observed phenomenon in a proper section.

We added more to the discussion on internal variation and other factors in the experiment, apart from amoxicillin, that may also have an effect on the microbiome (line 548-568)

L255: The increased abundance of *Cyanobacteria* was observed in control group but not in treatment group. Please refer to it in Discussion section. Take into account: May amoxicillin reduce growth of that phylum? To what kind of consequences it may lead to? What is the role of this phylum in human health or disease?

This is described in line 288 to 291, and more has been added to the discussion (line 502-505).

Discussion:

L 329-330: Please describe what kind of clinical effect (gut functionality or other) may be observed as a consequence of *Bacteroidetes*/*Firmicutes* changes.

This is elaborated in line 424-430.

L340: Phylum *Patescibacteria*: Please describe the role of that phylum if known, and provide the possible consequences for human health in case of significant reduction. It is important as in conclusion You highlight that this phylum never fully recovered. What are/ may be the implications?

This is described in line 438-443.

L 379: “specific alterations”: please specify exactly with referring to clinical consequences.

In this version of the manuscript we have assessed specific taxa further, and described our findings in the discussion.

L382: what kind of conditions initiated by microbiome alterations may persist for longer time? Please describe.

As mentioned above, in this version of the manuscript we have assessed the effect on specific taxa more thoroughly, and prolonged effects on certain ASVs/taxa are described.

Conclusion:

L 430: Further, even though overall diversity and composition appears to be insignificantly different from the original state, relatively soon after treatment, alterations in specific taxa, with **important functions or potential harmful effects may still occur** and should be assessed further.

You underlie important functions and potential harmful effect that may occur after antibiotic treatment, therefore in discussion section more potential implications of gut microbiome alterations should be described. Try to provide more hypothesis about possible clinical implications of microbiome changes observed in Your experiment.

The current version of the manuscript has been significantly altered in regards to specific taxa, which has improved the discussion on potential implications.
