# Supplementary material for: Effects of different amoxicillin treatment durations on microbiome diversity and composition in the gut
Source: PLoS One. 2022 Oct 27;17(10):e0275737. doi: 10.1371/journal.pone.0275737 (PMC9612567; doi:10.1371/journal.pone.0275737)
Supplement: Attachment — Submitted filename: Response to reiewers.pdf [file pone.0275737.s014.pdf]

Dear Juan J Loor, Academic Editor PLOS ONE

Thank you for the review comments that have now been taken into account in this revised version of the manuscript. In our response, we have given a point by point answer to the concerns and requests (marked in bold), as well as given the revised text in red italics with line and page numbers to the new version of the manuscript. We have included a "marked-up" separate file with track changes where all modifications to the manuscript has been made.

Kind regards
Hanne C. Winther-Larsen.

Response to Reviewer #1.

Reviewer #1: Thanks to the authors for addressing the vast majority of my comments, even if only to state them as limitations of the current study and important considerations for future work.

One important piece of information that I could not find in the manuscript that MUST be included prior to publication is whether or not rarefaction was utilized for any/all analyses and to what number of reads all samples were rarefied. I see in supplementary table 1 that there were adequate read depths, but readers will still need to know the methods used for analyses (i.e. rarefaction depth).

*In a previous version of the manuscript we had stated our rarefaction/rarefying analysis as resampling. This information is clarified in line 215-2017. It now reads "*The ASV dataset was normalized by rarefying to the sample with the lowest number of ASVs (= 18529 ASVs), using the *rarify_even_depth* function in the Phyloseq package (ref 54)."

I do hope that authors follow through with their statement to deposit corresponding data and metadata in a publicly available database.

The metadata has now been added with the manuscript as three supplementary data files and deposited in NCBI database with access no#: PRJNA875126*. This information is added in the manuscript;*

Line 627-630:

DATA AVAILABILITY STATEMENT

The datasets generated for this study can be found in the supplementary files ASV_all.csv, metadata_all.csv and taxonomy_table_all_2.csv, and is deposited at NCBI with accession numbers: PRJNA875126 and SAMN30602556-SAMN30602715.

Line 926-927.
Additional files encompass three supplementary tables containing the datasets generated for this study: ASV_all.csv, metadata_all.cvs and taxonomy_table_all_2.csv.
